# Municipal Contagious Disease Hospitals—Their Medical and Financial Importance to the Community1Read at the Buffalo Academy of Medicine, Tuesday evening, October 13, 1906

**Published:** 1908-11

**Authors:** R. J. Wilson

**Affiliations:** New York; Willard Parker Hospital


### Municipal Contagious Disease Hospitals—Their Medical and Financial Importance to the Community.[1]

By R. J. WILSON, M.D., New York.

Willard Parker Hospital.

IN considering questions of this kind we must have before us some example for illustration. I have taken as such example the contagious disease hospitals of the Department of Health of the city of New York that are under my supervision.

Their points of excellence can well be copied by other municipalities; their faults in construction and administration, when known, can be avoided. Some of these, both the good and the bad, I will call to your attention later in this paper.

Let us consider the medical importance of contagious disease hospitals from the following standpoints: first, the treatment afforded in them; second, the protecting influence of their rigid quarantine; third, their proper construction.

When we attempt to compare the treatment received by a patient at home with that received in a hospital, we are at once confronted with the question of home environment as compared with that of the institution. For the sake of argument we will grant that where good medical attention can be given and medicines, food and nursing are to be had as needed, the home is the better place for the patient. All sick persons, I take it, do better where they are best satisfied with their surroundings, and when they are taken from home to a hospital there is for a short time, a feeling of homesickness added to their other trouble. It so happens that the largest percentage of contagious disease occurs among that part of our population having the smallest amount of means, who are not able to give to the sick ones that amount of attention they really need and can not—on account of having to earn their daily bread day by day—protect their other children against infection.

You can compare, if you will, a tenement house apartment of three or four rooms in which are housed a family of five persons, with the clean and well ventilated wards of a modern hospital. In such a household the mother has the care of the sick child. Her day begins with the preparation of the early morning breakfast of the father, who, if allowed to work, must have this attention, then there are the other children to be dressed and cared for and kept away from the infection; the mother is in and out of the room where the sick one is at frequent intervals. She can not change her dress and use antiseptics in an intelligent way to protect her other children. The sick child, it is true, has

1. Read at the Buffalo Academy of Medicine, Tuesday evening, October 13, 1906

the mother's best care, but under the disadvantageous surroundings, this care is not as good as that afforded by a good nurse in a well regulated institution. In the hospital there is constantly on call the best of medical aid and the food and medicines are given with regularity. Sudden changes in the patient, and they come suddenly in contagious disease, are met at the onset and thus serious sequela are frequently avoided.

Let us consider here three of the most common of contagious diseases; first, diphtheria. What advantages does the hospital afford that can not be had in the home? There are croup cases that must be intubated; in the hospital there are experienced intubators capable not only of quickly and safely intubating, but what is much more to the point, know *when* intubation is indicated and when it is not. Unnecessary intubation is a useless and sometimes fatal operation for the patient, all of which is known and acted upon in the hospital.

How many physicians are there in this city would like to take the responsibility of intubating a case of diphtheria? How many physicians are there here who would like to assume the greater responsibility of leaving an intubated case at home where he knows that if the tube is coughed up, death will result if it is not speedily replaced? How many physicians are there who will assume the responsibility of holding croup cases out of a proper hospital and letting them die from neglect of a therapeutic which is so simple that not to take advantage of it seems almost criminal? I sincerely believe that intubation for croup should never be practised outside of a hospital unless the intubator can be called at a moment's notice and that well qualified trained nurses are in charge of the cases.

Diphtheria patients should have noses and throats regularly irrigated if they are to get the best modern treatment. This irrigation must be regularly and properly performed if we are to get the best results from it. I do not believe that proper irrigation can be had, except in a very few instances, outside of hospitals having good and specially trained nurses for this work. I make this statement only after having received many letters from hospitals, and physicians throughout America on the subject. If you need further evidence of the good effect of hospital treatment in diphtheria, compare the death-rate of Buffalo with that of Boston where over 60 per cent. of the cases are taken care of in the South Department of the Boston City Hospital. In Buffalo the death-rate being over 13 per cent., while in Boston it was less than 3 per cent. How can this wide difference be explained except by accrediting a great deal of it to the treatment afforded by the hospital. This seems logical, too, when we consider that every indication for antitoxin and other treatment is immediately responded to.

The care of scarlet fever in a hospital gives the patient a better chance to avoid the painful and dangerous sequela that frequently accompany it. I refer to the joint, gland and heart lesions. With the beginning of convalescence too often at home the patient is allowed to get out of bed and roam around the house. In the hospital the patient is kept in bed, the temperature is watched, the urine is examined and every guard is employed to prevent these sequelæ. The same thing may be said of measles. By constant watching by trained observers the dangers of sequela are lessened.

As important as the treatment of the contagious disease case and its ultimate recovery is the means used to prevent the disease spreading through the community. The regulations of the Department of Health of the city of New York are such that contagious diseases can not be discharged from the hospitals until it is believed that the contagious period in the case has passed. For example, scarlet fever is held for a minimum period of thirty-five days, and longer if the case is still desquamating; measles for twenty-one days and diphtheria until two negative cultures are returned. Thus it will be seen that convalescent cases are not allowed to come in contact with well persons and the spread of disease is prevented.

I have already said that hospital environment is the proper kind for contagious diseases, but this depends entirely upon the construction of the hospital and the manner in which it is administered. The Willard Parker Hospital for diphtheria, in the Borough of Manhattan, is a good type for the care of that disease. The principal points in it, to which I wish to draw your attention, are the admission and discharge rooms and the dressing rooms off the wards. When a case is brought to this hospital it is taken into the admission room immediately. This room has in it a table for examination, a medicine closet, a washstand and a stand and tray for the intubation set. There is adjoining it a bath-room. This admission room is in duplicate, so that in the event of a mistake in diagnosis or a case of mixed infection having been admitted, there will be another room to use while the infected one is being fumigated.

When the case is received in the admission room it is examined, the diagnosis made or confirmed, the patient receives a proper dose of antitoxin, and if not very sick is vaccinated. Cultures are made from the nose and throat and smears from the vagina of female children and sent to the diagnosis laboratory. It is taken into the bath-room, there divested of all its clothing and given a bath. It is then given an outfit of ward clothes and taken to the ward to which it has been assigned. The clothing worn by the patient on admission is sent to the disinfecting station there sterilised by steam and returned to the clothing room in the hospital where patients clothes are stored. When the patient is ready for discharge it is brought to the discharge room of the hospital, divested of its ward clothes, passes out of this room into a bath-room, which is situated between it and another room opening on the street. After receiving a bichloride bath and shampoo the patient passes into the room opening on the street, there receives its sterilised clothing, and after dressing is allowed to go out into the street as near aseptic as a human being can be made.

Off of the wards in the Willard Parker Hospital there are dressing rooms in which cases are extubated, or in which intubation is done when necessary. Taking cases into these rooms for these operations does away with a great deal of confusion in the wards that formerly accompanied them. Another feature of this hospital to which I wish to call attention is the stalls in the wards into which are placed the beds of patients suffering with broncho-pneumonia. These stalls are so arranged that ventilation is obtained without draughts. They are made of glass, so that nurses can see the patients at all times and from any part of the ward. It is probable that in the construction of a new building a series of small glass wards could be made that would be better than these stalls.

There is also in the Willard Parker Hospital a ward on the outside of the main building that is used exclusively for children that have vaginitis. Female children upon admission are sent to an observation ward until the report from the vaginal smear made upon admission is returned. This takes about an hour in the day time, but all cases admitted at night must be held until the following morning in the observation ward.

For the care of other contagious diseases, the Reception Hospital of the Borough of Manhattan offers a good type. This building is one story high, rectangular in shape, and has a solid partition running its whole length. It has five small wards on the north side and the same number on the south side. Each of these wards open directly into the outside air. There is not a hallway in the building. Each ward has its separate bath, lavatory and closet. There is a small area at the entrance to each ward, in which are kept the caps and gowns of the attending physicians and their assistants. This being an old building there are many things about it that can be improved upon, but the general principal is right. There could be glass partitions so that one nurse could attend more patients. We have in the Kingston Avenue Hospital in the Borough of Brooklyn these glass partitions, but, unfortunately, patients can not be taken into or removed from the rooms which they divide without exposing and being exposed to other patients. Such a condition is entirely wrong and should be avoided by others who can profit by our mistakes.

An ideal arrangement in hospital construction for contagious disease would be to have a series of admission rooms, one for each day, so that all patients admitted each day could be held until after the period of incubation of the various infections to which they might have been exposed, and after this period had passed be sent from these rooms to the general wards of the hospital. Such rooms having glass partitions and constructed on the plan of the Manhattan Reception Hospital would not add materially to the cost of administration and would surely prevent a great deal of costly mixed infection.

Visiting being a necessary evil of contagious disease hospitals, especially where the cases are dangerously ill, provision must be made for the proper control of visitors; first, to insure that they will not bring new infection into the hospital, and, second, that they do not carry out of the hospital infectious organisms to other persons. The construction of the wards of the Pasteur Institute is particularly adapted for this purpose. The patients are kept in small glass rooms and the visitors are admitted to a corridor where they can see the patients but can not come in contact with them. Our present method in New York of allowing gowned and capped visitors to pass into the wards and come in actual contact with the patients is unscientific and without a single good feature to warrant it.

Let us now leave the purely medical side of this subject and consider briefly the elements that enter into the cost of caring for contagious diseases in hospitals that makes them more expensive than those for noncontagious diseases. First comes the cost of isolation. You may have but one case of a certain infection, but if properly cared for it must be put in a ward or room by itself and a day and night nurse assigned to it exclusively. In a hospital for noncontagious diseases this would represent nurses enough for ten cases if they were not seriously ill. Not only have we the cost of the nurses but also the ward helper; she cannot go from one service to another indiscriminately. Then there is the cost of caring for the various articles used in the rooms, the disinfection and sterilisation that must be constantly practised; the patient's clothing and the laundry must be sterilised and extraordinary care must be used in handling the infected articles that have been in contact with the case. The isolation and care attendant upon it necessitates a much larger pay roll than is necessary in a general hospital, and of course, the cost of maintenance is in direct ratio to the size of the pay roll.

If it costs $2.50 a day to care for a patient in a general hospital, and $3.50 to $5.00 a day to take care of a case in a contagious disease hospital, it certainly would seem that a contagious disease hospital was a very expensive proposition as compared with others; if it costs $3.50 a day to take care of a case in a contagious disease hospital and only $1.50 to take care of the same case at home it certainly looks as if it would be a saving investment to keep the case at home, and so it would if the expense ended with the care of the case but it does not and right there is the reason for having contagious disease hospitals.

Let us consider first the school time forfeited by children in a family where there is a case of contagious disease,—scarlet fever for example. Every child of school age in this family must be kept out of school for a minimum period of five weeks. Here you have a direct loss in money. In Buffalo, roughly speaking, the per diem amount paid for each child is ten cents, then for every child quarantined the direct money loss is about three dollars. Last year you had over 900 cases of scarlet fever: this cost you in school time lost $2,700, this could not be avoided even if the cases had been cared for in a hospital but if there were say two other children of school age in each family, you had a total and unnecessary loss of $5,400. You had nearly 3 000 cases of measles. The total loss to the city on the same basis would be about $6,000. In this disease the quarantine is only three weeks.

Diphtheria with an average quarantine of ten days, would have cost you $500. On school time you had in 1907, a loss of nearly $12,000, which would go a long way toward maintaining a contagious disease hospital. But that is not the greatest loss, many of these quarantined children get so far back in their studies that they are obliged to take the whole course over again, and here you have the loss of a whole school year which is a trifle over $30 in money lost to the city and infinitely more to the child, who has lost the time. Say that 5 per cent. of the children lose the whole year as a result of this quarantine, you have in scarlet fever alone a loss of nearly a $1,000 dollars.

I fully recognise the fact that it is possible to make a great show with figures if you wish to prove your point. But here you have the facts to work on; every taxpayer in Buffalo ought to know what is the total amount paid out for schools; he can easily find out the number of children in the schools and the cost of caring for each child; it is then easy enough to figure the loss in school time. I am making these estimates roughly but anyone here interested enough can get at the exact facts.

Besides the loss in school time, which can be figured pretty accurately, we have also to consider the loss through quarantine of business premises. In New York City a good percentage of cases in the contagious disease hospitals come from such places. If no provision is made to take care of contagious disease cases that occur in stores, shops, dairies, and the like, they must, of course, be closed during the period of quarantine. It is impossible to estimate the exact money loss of such quarantine. I can best illustrate it by calling your attention to one of many cases that came under my observation last winter. The Willard Parker Hospital ambulance station received a call in the late evening to remove a case from a house in one of the downtown streets. For some unknown reason this call was not immediately responded to and a few hours later I was appealed to by the father of the child to hurry up the ambulance and get the case. This man was a barber, occupying the ground floor of a business building; his shop being in the front and his dwelling in the rear. He employed several men, and made a living for himself and furnished employment to the others, so that his shop represented the earning capacity of several men. Now the regulations of the Department of Health of the city of New York, very justly, do not allow business to be continued while a contagious disease is in the same apartment. This business man told me that he must keep his shop open in order to meet his expenses. Hence his anxiety to get his child out of the house and into the hospital. I have no doubt that somewhat similar examples could be shown by going over the files of the Health Department of this city.

In New York city if every store and shop was closed where contagious disease appears, it would represent thousands of dollars of lost time of the persons employed in them. Under the conditions that now exist the children are taken to the hospital, the infected area is disinfected and within a few hours from the reporting of the case business is proceeding in the regular way. I might also say that people as a general rule do not care to trade in a store or send their children to it on errands, if they know there is a contagious disease on the premises.

In many instances the members of the family are not allowed to carry on their usual occupations if there is a contagious disease in the house. School teachers, dairymen, and the like, represent this class. Again, hotels and boarding houses must cease to do business while the case of contagious disease is present. All this represents a loss that can not be estimated, but it is a safe guess that could it be calculated it would be found that the money thus totally lost would be sufficient to support a hospital that would easily take care of a like number of cases.

It is a fair proposition that in the long run a contagious disease hospital is a paying investment for any municipality. And right at this point I would like to say that there is no reason why there should not be paying wards and private rooms in the hospital. You will find that as people become more acquainted with hospital care they will be only too glad of an opportunity to pay to have their children sick with contagious disease taken to the hospital, and thus be relieved of the responsibility of preventing the spread of the disease in their own homes first, and then through the community while at the same time conscious of the fact that they are giving them the very best care that it is possible to command. While we have these examples of direct loss to the community as a result of no proper place to take care of contagious diseases, the establishment of a hospital means money saved through preventing the spread of infection and non-interference with the routine of business. You have all noticed that when people have been notified that a blast has been put in and is about to be exploded they keep out of the way and avoid injury. It is very much the same thing with the contagious disease hospital; the cases are all gathered together in one place, everybody knows they are in that place, and the people taking care of them make it a point to see that no contagion spreads from the hospital.

Last year a virulent attack was carried on against the Kingston Avenue Hospital for contagious diseases for a long time in an effort to have it removed from its present site. The argument used was that it was a menace to the health of the immediately surrounding community. Upon investigation it was found that there had not been a case of contagious disease within several blocks of the hospital for a long time and that no member of the families of the 200 employees had had contagious disease as a result of their working there, although at that time there were 500 cases of contagious disease in the hospital. What better argument can be given for the good to the community of a contagious disease hospital? Have your own records examined and find out how each case in families kept at home becomes the focus for several other cases. People walk in on these local cases unawares, tradespeople help to spread the disease, innocently it is true but none the less surely.

Every time a case of contagious disease is taken to a hospital it means that a focus for the possible spread of the disease has been removed. There is no way of estimating how much has been saved to the community by this, but it is safe to assume that what is saved in one year would be sufficient to build and administer a hospital for that year and the savings of following years would be like an accumulating surplus.

In closing I wish to say a word about the social relation of the contagious disease hospital to the community. The old appellation of pest house, coupled with ignorance on the part of the people, has caused a tremendous amount of expense and distress. Pest houses bring to mind loathsome things, dangerous things, things to be avoided, things to be afraid of. Now contagious diseases are not loathsome and are not more dangerous, when properly administered, than other communicable diseases. It is our business as physicians to tell our clients this, always qualifying our statements with the caution of proper care; and in no instance can better care be had than in a well regulated hospital.

It is not necessary to hunt for some secluded spot for a hospital: on the contrary, it should be so centrally located that the ambulance calls would be the shortest possible. It does not help a very sick case to be transferred long distances by ambulance. Manage the contagious disease hospital right and it can be placed in the most densely settled part of the community without danger to anyone. Look at the Willard Parker Hospital, New York, in one of the most thickly settled parts of the city and one in which there is but little contagious disease as compared with some other parts of the city. A city the size of Buffalo without a proper place to care for its contagious diseases is away behind the march of civilisation both scientific and humanitarian.

